# Serum leptin levels in Iranian patients with Parkinson's disease

**Published:** 2018-04-04

**Authors:** Mehri Salari, Mahdi Barzegar, Masoud Etemadifar, Omid Mirmosayyeb

**Affiliations:** 1 Department of Neurology, School of Medicine, Isfahan University of Medical Sciences, Isfahan, Iran; 2 Neurosciences Research Center, Alzahra Research Institute, Isfahan University of Medical Sciences, Isfahan, Iran; 3 Student Research Committee, School of Medicine, Isfahan University of Medical Sciences, Isfahan, Iran

**Keywords:** Parkinson Disease, Leptin, Iran, Parkinsonism

## Abstract

**Background:** Parkinson's disease (PD), the second most prevalent neurodegenerative disorder, has been related with weight loss and energy balance. Some studies showed that leptin might be playing an important role in satiety, energy balance and immune response. The aim of this study was to evaluate serum leptin level in patients with PD and its association with clinical severity.

**Methods:** In this cross-sectional study, 35 patients with PD and 51 healthy controls (HCs), matched for age, sex and body mass index (BMI), were recruited. Serum leptin level was measured and clinical characteristics and demographic data of patients were recorded.

**Results:** The mean age of patients with PD and HCs were 59.80 ± 11.40 and 62.18 ± 11.60 years, respectively. Serum leptin concentration was not statistically different between patients with PD and HCs (21.1 ± 23.1 ng/ml vs 25.9 ± 21.8 ng/ml, P = 0.280). There was no relation between plasma level of leptin and clinical severity of patients with PD.

**Conclusion:** Our findings suggest that serum level of leptin is neither implicated in the pathogenesis of PD, nor decreases as disease progresses.

## Introduction

Parkinson’s disease (PD) is the most frequent movement disorder that commonly affects elderly people.^   1 ^ Initially, PD was known as a motor disorder with principal symptoms of bradykinesia, resting tremor, rigidity and postural instability.^   2 ^ In the last decade, several studies have emphasized on impact of non-motor symptoms (NMS) such as depression, constipation, pain and sexual difficulties in progression of disease and patient’s quality of life (QOL).^3^^-^^6^ Some studies demonstrated that patients with PD have weight loss and reduced body mass index (BMI).^7^^,^^8^ Nevertheless, the cause of these events is not clear.

Leptin is a long acting endocrine peptide hormone produced by adipose tissue. Moreover, a small amount of leptin secretes from brain. Leptin controls energy balance through suppression of food intake, glucose metabolism and energy expenditure.^   9 ^ Leptin crosses the blood-brain barrier (BBB) and stimulates hypothalamic receptor and sympathetic pathway, resulting in controlling energy intake and energy expenditure.^   10 ^ Previous studies found leptin serum levels in patients with PD lower than that in healthy controls (HCs), though the correlation of weight loss and leptin levels was not confirmed.^        11 ^ Furthermore, previous studies revealed that patients with PD have lower BMI and lower serum concentrations of leptin compared with HCs. Nevertheless, there is not significant association between serum concentrations of leptin and BMI, and this correlation is not clear exactly.^12^^,^^13^ 

Animal and human studies demonstrated the regulatory function of leptin in nervous system and showed that non-motor manifestations such as cognitive dysfunction of patients with PD as well as mesostriatal and mesolimbic dopaminergic pathways are affected by serum and cerebrospinal fluid (CSF) level of leptin.^14^^-^^18^ On the other hand, both motor symptoms and NMS influence the energy intake and energy expenditure.

We preformed the present study to evaluate leptin profile in patients with PD and assess the possible relation between motor symptoms and NMS of patients with serum level of leptin.

## Materials and Methods

We enrolled thirty five eligible patients (25 men and 10 women) with idiopathic PD (IPD) who were admitted to Al-Zahra hospital, Isfahan, Iran. Fifty one healthy people (28 men and 23 women), matched for sex and age, were studied as control group. The study was approved by the regional bioethics committee of Isfahan University of Medical Sciences, and written informed consents were obtained from all patients prior to enrollment.

All patients were visited by a neurology specialist in movement disorders, and diagnosis of PD was stablished based on United Kingdom Parkinson Disease Society Brain Bank (UK-PDS-BB) criteria.^        19 ^ The subjects who were unable to ambulate, patients with systemic diseases including endocrine and malignant diseases, and those with psychiatric disorders (psychosis and severe depression) were excluded. Moreover, patients treated with medications influencing nutritional state and/or BMI such as corticosteroids, antihistamines and antipsychotic at least for six months, were excluded. None of the patients had nausea or anorexia due to dopaminergic medication and their dietary habits did not alter during the study.

Motor symptoms and NMS of patients with PD were assessed using unified Parkinson’s disease rating scale (UPDRS) parts I, II, III and IV. Clinical status of the patients was evaluated by Hoehn and Yahr scale (HY scale). Cognitive state of patients was surveyed using Mini Mental State Examination (MMSE). In addition, BMI of patients and HCs were calculated.

A single 3ml venous blood sample was collected from all patients. Serum was separated within 30 minutes and sorted at -80 ^∘^C until analysis for leptin. Serum leptin levels were measured by a leptin radioimmunoassay (RIA) (Linco Research Ltd., St. Charles, MO, USA) and all samples were analyzed in duplicates in the same assay.

All statistical calculations were done using SPSS software (version 20, IBM Corporation, Armonk, NY, USA). Data were expressed as means ± standard deviation (SD), median, and frequency. The one-sample Kolmogorov-Smirnov test was performed for checking variables normality. The between group differences and correlation were assessed using independent sample t-test, Mann-Whitney U test, Spearman and Pearson’s correlation, respectively. Statistical significance (P value) was set at the level up to 0.05.

## Results

Table 1 demonstrates demographic features and clinical characteristics in patients with PD and HCs. The one-sample Kolmogorov-Smirnov test showed normality of variables (K-S = 1.354, P = 0.051). 

**Table 1:** Demographic profiles and clinical characteristics of patients with Parkinson’s disease (PD) and the healthy controls (HCs)

**Variables**	**Patients with PD**			**Controls**		
	**Total**	**Men**	**Women**	**Total**	**Men**	**Women**
Age (year) (mean ± SD)	59.80 ± 11.40	61.30 ± 11.10	56.20 ± 12.40	62.18 ± 11.60	63.50 ± 12.30	58.90 ± 10.10
Weight (kg) (mean ± SD)	66.10 ± 8.70	66.50 ± 9.20	65.20 ± 8.10	67.10 ± 10.10	79.10 ± 13.20	71.80 ± 11.50
BMI (kg/m^2^) (mean ± SD)	24.80 ± 3.40	24.40 ± 3.50	25.80 ± 3.50	25.00 ± 3.20	27.00 ± 4.90	27.70 ± 5.10
MMSE (mean ± SD)	26.80 ± 3.30	27.1 ± 3.60	24.80 ± 4.00	-	-	-
Duration (year) (mean ± SD)	4.20 ± 3.60	3.75 ± 4.14	3.60 ± 1.51	-	-	-
HY scale (mean ± SD)	1.80 ± 0.90	1.75 ± 0.80	1.80 ± 0.80	-	-	-
UPDRS total (mean ± SD)	49.10 ± 26.80	51.10 ± 25.80	39.80 ± 17.00	-	-	-

PD: Parkinson’s disease; BMI: Body mass index; MMSE: Mini Mental State Examination; HY scale: Hoehn and Yahr scale; UPDRS: Unified Parkinson’s Disease Rating Scale

Association of IL-6 level with NIHSS, mRS and other infarcts

**Figure 1:**
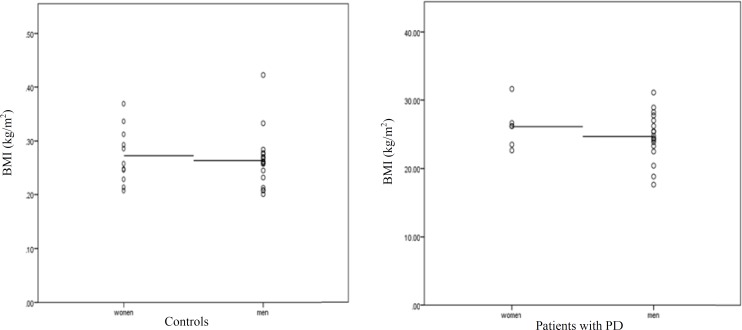
Patients with Parkinson’s disease (PD) and controls participants presented similar body mass index (BMI)

Figure shows mean and standard error of mean.

46.2% of patients with PD were at stage 2 of HY scale, and 26.9% and 15.4% of patients were at stages 1 and 3, respectively. The mean scores of UPDRS parts I, II and III were 3.5 ± 3.7, 13.1 ± 8.1, and 30.5 ± 17.7, respectively. No patient suffered from severe disability and nobody were bed ridden. Moreover, patients with PD had similar BMI index to HCs (Figure 1).

**Figure 2:**
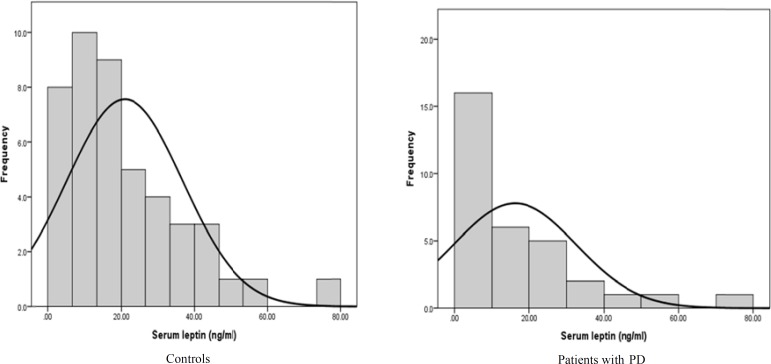
Histogram of leptin levels in patients with Parkinson’s disease (PD) and healthy controls (HCs)

In Figure 2, frequency distributions were shown. Serum concentration of leptin in patients with PD and HCs were 21.1 ± 23.1 and 25.9 ± 21.8, respectively.

**Figure 3:**
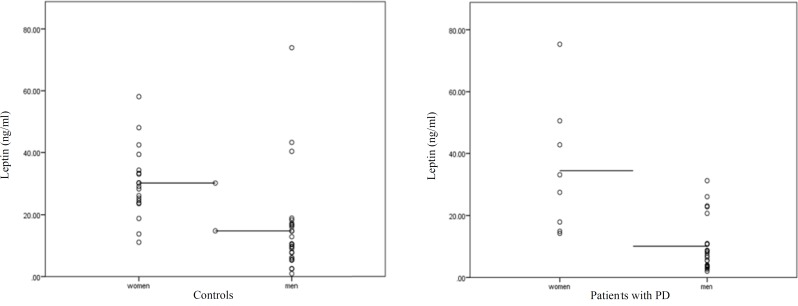
Patients with Parkinson’s disease (PD) and healthy control participants had similar leptin levels. Figure shows mean and standard error of mean.

Although serum level of leptin in patients with PD was lower than that in HCs, it was not statistically significant (Figure 3). Male patients had significant lower plasma leptin concentrations compared with female patients.

Among patients with PD, correlation of leptin level with age, weight and duration of disease was analyzed. The leptin levels in male patients was significantly correlated with duration of disease (r = -0.481, P = 0.043). Moreover, leptin levels had significance correlation with BMI in all participants (r = 0.326, P = 0.017).

The correlation between leptin, BMI and clinical features, including HY scale, UPDRS parts I, II, III, IV, and total score, MMSE, and duration of disease was evaluated. 

The UPDRS part I had significant correlation with leptin level in male patients (r = -0.625, P = 0.050) and female patients (r = -0.817, P = 0.023). In contrast, UPDRS part II correlated with leptin in female patients only (r = -0.725, P = 0.042). Moreover, leptin levels had significant relationship negatively (r = -0.489, P = 0.010) with duration of disease in male patients. The total UPDRS score and UPDRS part II were significantly higher in patients with low BMI (P = 0.031 and P = 0.041).

## Discussion

This study examined leptin serum levels in patients with PD comparing with HCs. To the best of our knowledge, it is the first study to investigate leptin levels in Iranian population with PD. Our results showed no significant difference of serum leptin levels among patients with PD compared to HCs. However, leptin levels associated with sex and BMI. 

Such results support other studies which showed that serum level of leptin in patients with PD was not different from controls.^11^^,^^20^^-^^23^ Rocha, et al reported that adipokine levels in advanced PD patients were not different with age-gender-BMI-matched controls and had no association with clinical findings in patients with PD.^        20 ^ These finding were consistent with results of another study with limited sample size (eight PD patients and eight controls) that authors assessed adipokine levels in early PD patients. They found no difference in levels and diurnal rhythm of leptin in patients with PD and concluded that weight loss and neurodegenerative pathways in PD have no association with adipokine secretion.^   21 ^ Ozdilek and Kenangil showed that leptin levels correlated with age, gender, and BMI, but disease duration and clinical severity had no effect in leptin concentration. They suggested that leptin levels have no role in progression of disease.^   22 ^ In our study, we found no significant difference between plasma leptin levels in patients with PD and HCs.

Previous studies surveyed leptin levels in PD patients and its relation with unintended weight loss. They reported that serum levels of leptin were lower in PD patients compared to HCs. Furthermore, they found that PD patients with unintended weight loss had lower leptin levels.^12^^,^^13^ Serum leptin concentrations correlate to body fat mass in PD patients with and without decreased weight and HCs.^11^^,^^12^ Moreover, Pelleymounter, et al revealed that leptin associates with body fat and weight loss by decreasing food intake and increasing energy expenditure.^        24 ^ These results express the hypothesis that the cause of leptin levels abnormality in PD patients maybe reduction of body fat mass. 

Several studies surveyed the correlation of patients’ age with serum leptin levels and demonstrated that elderly PD patients have higher leptin levels, especially men.^25^^,^^26^ There is no exact reason for this finding. Soluble leptin receptor (Ob-Re) level does not depend on gender and this data support that leptin axis in healthy population associated only with aging.^             27 ^ Our results confirm previous findings regarding leptin association with age in PD patients.

In agreement with previous studies, gender differences relate to leptin levels. Several studies in recent years have shown that leptin levels are lower in male PD patients in comparison with female ones. There was abundant evidence to prove roles of increased proportion of adipose tissue and increased production of leptin per body fat mass unit in this correlation.^11^^,^^28^ Moreover, such studies suggest association between androgenicity in individuals with leptin levels that support gender differences in serum leptin level.^29^^,^^30^

The present results remain in agreement with the majority of published data showing a progressive increase in the leptin serum levels with an increase in BMI in participants.^21^^,^^22^ In these studies, a leptin level was not different between PD patients and HCs, but leptin level correlated with BMI. 

Association of leptin with BMI is reported in healthy population and other disorders including polycystic ovarian disease (PCOD) and rheumatoid arthritis (RA).^31^^-^^35^ Investigation on patients with PCOS reported that leptin concentration associated with BMI, and was not correlated with hormonal indices in PCOD patients. Moreover, leptin level was different between obese and non-obese women.^32^^,^^33^ In RA patients without any inflammatory disorders, leptin level correlated with BMI, although its levels were similar between patients and HCs.^34^^,^^35^ Deep brain stimulation (DBS) in the subthalamic nucleus (STN) caused weight gain in PD patients, but leptin levels did not change after surgery.^36^ These findings reinforce the hypotheses that leptin level alterations in some disorders such as PD associates with BMI and fat mass rather than disorder. The results and characteristics of all previous studies that investigated leptin levels in PD patients are summarized in table 2.

A limitation of our study and previous studies was the small sample size. We did not evaluate fat mass of participants, while it can affect circulatory leptin levels and clarify relation of leptin levels with PD.

## Conclusion

In the light of all previously stated facts, we can conclude that circulating leptin level in patients with PD is not different from healthy population and is not associated with disease severity, duration of disease, and clinical status.

**Table 2:** Studies that reported the leptin levels of patients with Parkinson’s disease (PD)

**Study**	**Patients ** **(n) (M/F)**	**Controls ** **(n) (M/F)**	**Participants’ age (year) ** **(mean ± SD)**	**Disease characteristics**	**Leptin patients ** **(mean ± SD)**	**Leptin ** **controls ** **(mean ± SD)**	**Outcomes**
Fiszer, et al.^12^	27 (13/14)	12 (3/9)	Patients: 61.70 ± 8.74	UPDRS: 40.00 ± 20.10 MMSE: 27.40 ± 2.40 Duration of PD (year): 7.20 ± 4.10 MADRS: 8.40 ± 7.10	6.10 ± 5.30	13.69 ± 10.05	leptin levels were lower in PD patients compared to controls, patients with weight loss had lower leptin in comparison to patients without weight loss, positive correlation between leptin and adipose content in all individuals
			Controls: 58.58 ± 8.60				
Evidente, et al.^13^	36 (WL: 18, WS: 18)	-	WL: 70 ± 2	83% of WL and 72% of WS were on L-dopa, perception of hunger and thirst and the sensation of fullness were similar in both groups	WL: 4.70 ± 1.20 WS: 6.30 ± 1.90	-	Weight is unlikely to be due to abnormal Leptin levels in PD, leptin levels had correlation with BMI in WL and WS patients
			WS: 64 ± 3				
Rocha, et al.^20^	40 (27/13)	25 (19/6)	Patients: 68.70 ± 10.00	UPDRS: 51.80 ± 25.20 MMSE: 24.00 ± 3.90 HY: 2.44 ± 0.69 SE-ADL: 77.95 ± 11.96%	1420.53 ± 469.29	1472.19 ± 341.83	PD patients and controls had similar leptin levels, leptin levels associated with BMI, no correlation between leptin levels and clinical or demographic data was found
			Controls: 65.20 ± 8.70				
Aziz, et al.^21^	8	8	-	De novo patients	12.40 ± 12.20	15.10 ± 11.50	Leptin levels in PD patients and controls were similar, Levels of leptin were not associated with disease severity, positive correlation between leptin levels and fat mass in patients and controls
Ozdilek and Kenangil^22^	40 (28/12)	25 (14/11)	Patients: 60.80 ± 9.40	UPDRS: 25.80 ± 15.60 HY I (35%), II (35%), III (35%), IV (35%) Duration of PD: 6.30 ± 4.50 LEDD: 714 ± 409	6.80 ± 6.90	3.90 ± 3.80	Leptin levels in PD patients and controls were similar, leptin levels had no correlation with disease severity, positive correlation between leptin levels and BMI, WC and BW in PD patients
			Controls: 61.80 ± 5.80				
Kenangil and Ozdilek.^23^	30 (25/5)	30 (22/8)	Patients: 59.37 ± 9.21	UPDRS total: 28.20 ± 18.40 UPDRS III: 13.6 ± 8.10 HY: 2.20 ± 0.90 LEDD: 777 ± 408 mg	4.13 ± 3.61	3.12 ± 2.43	PD patients and controls had similar leptin levels, no association between leptin levels and motor or functional impairment in PD, leptin levels had correlation with BMI, WC and BW in PD patients
			Controls:58.50 ± 9.85				
Lorefalt, et al.^11^	26 (9/17)	26 (9/17)	All participants were older than 60	UPDRS: 26.70 ± 17.90 14 patients newly diagnosed, 12 patients treated 5.00 ± 2.70 years with L-dopa	14.70 ± 9.20	18.70 ± 13.40	Leptin levels in PD patients and controls were similar, levels of leptin correlated to body fat mass in patients and controls

Numerical outcomes are expressed as means ± standard deviation (SD).

M/F: Male/Female; PD: Parkinson's disease; BMI: Body mass index; HY scale: Hoehn and Yahr scale; MMSE: Mini Mental State Examination; UPDRS: Unified Parkinson’s Disease Rating Scale; LEDD: Levodopa equivalent daily dose; MADRS: Montgomery-Asberg Depression Rating Scale; WL: Weight loss; WS: Weight stable; SE-ADL: Schwab and England Activities of Daily Living scale

## Notes:

**How to cite this article:** Salari M, Barzegar M, Etemadifar M, Mirmosayyeb O. Serum leptin levels in Iranian patients with Parkinson's disease. Iran J Neurol 2018; 17(2): 71-7.
